# Coronary heart disease and stroke mortality trends in Australia and New Zealand: comparison of official national mortality data and Global Burden of Disease estimates

**DOI:** 10.1093/ije/dyaf112

**Published:** 2025-07-02

**Authors:** Yuehan Zhang, Grace Joshy, Karen Bishop, Tim Adair, Wendy Ho, Katrina Sheehan, Michelle Gourley, Rod Jackson, Mai Nguyen, Emily Banks, Ellie Paige

**Affiliations:** National Centre for Epidemiology and Population Health, The Australian National University, Canberra, Australia; National Centre for Epidemiology and Population Health, The Australian National University, Canberra, Australia; Burden of Disease and Mortality Unit, Australian Institute of Health and Welfare, Canberra, Australia; Nossal Institute for Global Health, Melbourne School of Population and Global Health, The University of Melbourne, Melbourne, Australia; Burden of Disease and Mortality Unit, Australian Institute of Health and Welfare, Canberra, Australia; Burden of Disease and Mortality Unit, Australian Institute of Health and Welfare, Canberra, Australia; Burden of Disease and Mortality Unit, Australian Institute of Health and Welfare, Canberra, Australia; School of Population Health, Faculty of Medical and Health Sciences, University of Auckland, Auckland, New Zealand; National Centre for Epidemiology and Population Health, The Australian National University, Canberra, Australia; National Centre for Epidemiology and Population Health, The Australian National University, Canberra, Australia; National Centre for Epidemiology and Population Health, The Australian National University, Canberra, Australia; Population Health Program, QIMR Berghofer Medical Research Institute, Brisbane, Australia; School of Public Health, University of Queensland, Brisbane, Australia

**Keywords:** cardiovascular disease, age-standardized mortality rates, Global Burden of Disease

## Abstract

**Background:**

Recent Global Burden of Disease (GBD) estimates show increasing cardiovascular disease (CVD) mortality in Australia and New Zealand, prompting concern. This study investigates whether such increases are observed in official national data.

**Methods:**

Annual age-standardized coronary heart disease (CHD) and stroke mortality rates for ages 35–84 years in Australia/New Zealand from 2008 to 2018/19 were calculated using official national data and published GBD estimates. Differences in annual mortality rate percentage changes between official data and GBD estimates were calculated separately for each country. Joinpoint regression identified temporal trend changes.

**Results:**

Official data showed annual decreases in CHD mortality of 4.9% (95% CI: 4.4%–5.5%) for Australia and 4.2% (3.7%–4.7%) for New Zealand on average; corresponding annual stroke mortality reductions were 4.3% (3.2%–5.3%) for Australia and 3.7% (2.9%–4.5%) for New Zealand. Absolute CHD mortality rates from GBD were substantively higher than from official data (e.g. 104.7 [103.0–106.5] vs 99.0 [97.3–100.7] per 100 000 people, respectively, Australia, 2008). Contrasting with ongoing declining rates using official data, GBD estimates showed slower overall mortality rate declines, recent increases in CHD mortality (e.g. 1.2% [0.2%–2.2%] annual increases from 2016 to 2019 for Australia), and stagnating stroke mortality. Differences are likely explained by GBD’s redistribution of ill-defined causes of death and use of projected data after 2016, when national mortality data were unavailable for GBD estimates.

**Conclusions:**

CHD and stroke mortality in Australia/New Zealand continue to decline, according to gold-standard official data. Disparities with GBD estimates highlight the need for transparency in reporting GBD methods and care in interpretation and application.

Key Messages

- Concerns have been raised that cardiovascular disease mortality has stagnated or increased since 2013 in certain high-income countries, based on published Global Burden of Disease (GBD) estimates.
- Official national mortality data show ongoing declines in age-standardized coronary heart disease (CHD) and stroke mortality for both Australia and New Zealand from 2008 to 2018/19. In contrast, GBD estimates show higher absolute CHD mortality rates, recent increases in CHD mortality rates, and stagnation in stroke mortality rates.
- These disparities are likely due to GBD’s use of projected data from 2016 onwards, and the redistribution of ill-defined causes of death. Reliable, official statistics should be prioritized, and transparency in reporting methods should be improved.

## Introduction

Cardiovascular disease (CVD) is a leading cause of death worldwide and the underlying cause of 26% of deaths in Australia [1] and 33% of deaths in New Zealand in 2019 [2]. Mortality statistics are central to monitoring progress against CVD and making international comparisons. In Australia, official national mortality data show an 82% decline in CVD mortality rates since 1968 [3]. The most recent analyses of official national mortality data indicate ongoing declines in overall CVD mortality in Australia up to 2017, with some evidence of a recent slowing in this decline, which has also been observed in some other high-income countries [4, 5].

In Australia and New Zealand, although multiple causes of death are listed on death certificates, official mortality data focus on the underlying cause of death, referring to the primary condition initiating events leading to death [6, 7]. This approach supports consistent monitoring of conditions like CVD over time.

The Global Burden of Disease (GBD) Collaboration, which also uses the underlying cause of death to classify mortality [8], is a highly regarded initiative that synthesizes a large number of data sources to estimate mortality, causes of death and illness, and risk factors and provides comprehensive assessments of trends over time and by country. Data are processed—including for cause of death redistribution and correction for non-reference case definitions or measurement methods—and modelled using standardized tools including Cause of Death Ensemble model (CODEm), spatiotemporal Gaussian process regression, and DisMod-MR to generate estimates of each measure of interest by age, sex, location, and year [8]. Processes may account for lags in availability of official vital registrations data. Concerns have been raised regarding a lack of transparency in GBD imputation and estimation methods, which can diverge considerably from country statistics [9].

GBD 2019 estimates showed decreases in age-standardized CVD mortality rates from 1990 to 2013 for Australia and New Zealand and slight increases from 2013 to 2019 [8]. However, since official cause-specific mortality data were only available up to 2015 (New Zealand)/2016 (Australia) at the time of publishing, GBD estimates for 2016/17–2019 were projections rather than observed data.

The suggestion of stagnating or increasing CVD mortality raises serious concerns, particularly for chronic disease policy and practice, and achievement of non-communicable disease goals [10]. To ascertain whether such trends are truly present and to inform best practice, we calculated coronary heart disease (CHD) and stroke mortality rates and trends over time in Australia (2008–19) and New Zealand (2008–18) using currently available official national mortality data, and compared these with equivalent GBD estimates.

## Methods

The main outcomes were deaths from CHD (International Statistical Classification of Diseases and Related Health Problems 10th edition [ICD-10] codes: I20–I25) and stroke (ICD-10 codes: I60–I69 and G45–G46) [11], consistent with GBD definitions.

### Data sources and study population

#### Official national mortality data

We extracted summary data on causes of deaths in Australia and New Zealand, from the General Record of Incidence of Mortality (GRIM) books published by the Australian Institute of Health and Welfare (AIHW) [12], and from the New Zealand Ministry of Health Mortality Collection [13], respectively. Data extracted were from 2008 to 2019 for Australia and 2008 to 2018 for New Zealand for people aged 35–84 years at time of death, and included number of deaths and crude rates, by 5-year age group, sex, and death registration year. Official national mortality data did not include redistribution of ‘ill-defined’ causes of death. The GRIM books did not include ICD-10 codes G45 and G46 in the definition of mortality from stroke in Australia. However, sensitivity analyses comparing previously reported stroke deaths for all ages in 2019 indicate negligible likely effects (number of deaths 0.7% lower when excluding G45–G46) [14].

#### GBD mortality estimates

Corresponding GBD estimates of number of deaths and crude rates were extracted using GBD Vizhub, a data visualization tool [15]. GBD 2019 causes of death data in Australia and New Zealand are reported as being based on vital registration, provided by the Australian Bureau of Statistics (and sourced from the World Health Organization Mortality Database) and the New Zealand Ministry of Health; latest year of input data was 2016 for Australia and 2015 for New Zealand [16, 17]. Unlike official national data, GBD estimates for Australia and New Zealand for years 2017 (Australia)/2016 (New Zealand) onwards are therefore based on out-of-sample predictions. Additionally, GBD data processing redistributes ‘ill-defined’ causes of death (e.g. heart failure [ICD-10 code I50]) to causes with greater policy utility [8, 18].

### Statistical analyses

Crude mortality rates for CHD and stroke, from the official national mortality data and GBD, were directly standardized to the GBD 2019 population [19, 20], in 5-year age groups (Supplementary Table S1). For each country and outcome, age-standardized rates over time and corresponding 95% confidence intervals (95% CIs), assuming binomial proportion, were calculated and plotted by data source [21], with differences in mortality rates compared using the Wald test.

Joinpoint regression was used to identify statistically significant change in trends over time [22], with Weighted Bayesian Information Criterion [23] used to select the optimal number of joinpoints. Average annual percent changes between joinpoints and for the full study period were reported.

Post-hoc analyses included: (i) calculating age-standardized mortality rates for CHD and stroke separately for males and females; and (ii) investigating the impact of redistribution of ill-defined causes of death by examining a composite outcome of all circulatory system diseases (ICD-10 codes I00-I99, hereafter referred to as ‘all CVD’). The latter analysis was restricted to Australia as we did not have access to data on all CVD for New Zealand.

Data analyses were done using Excel and the Joinpoint Regression Program, Version 5.2.0.

## Results

### CHD mortality rates in Australia

**Figure 1.**
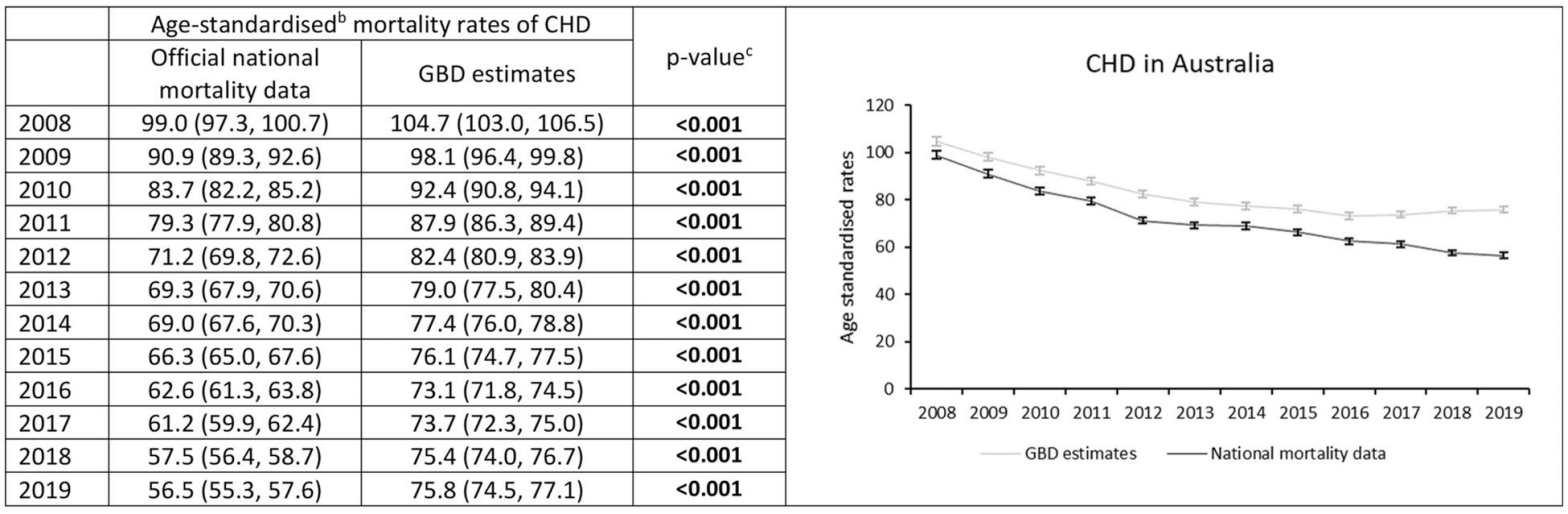
Coronary heart disease mortality rates from 2008 to 2019, estimated from official national mortality data and the Global Burden of Disease study for the Australian population aged 35–84 years. ^a^Coronary heart disease (CHD) defined according to International Classification of Disease codes of I20–I25. ^b^Mortality rates are age-standardized to the Global Burden of Disease (GBD) Study World Standard Population. ^c^*P*-values were calculated using Wald test, with values less than 0.05 indicating a significant difference between the data sources.

**Figure 2.**
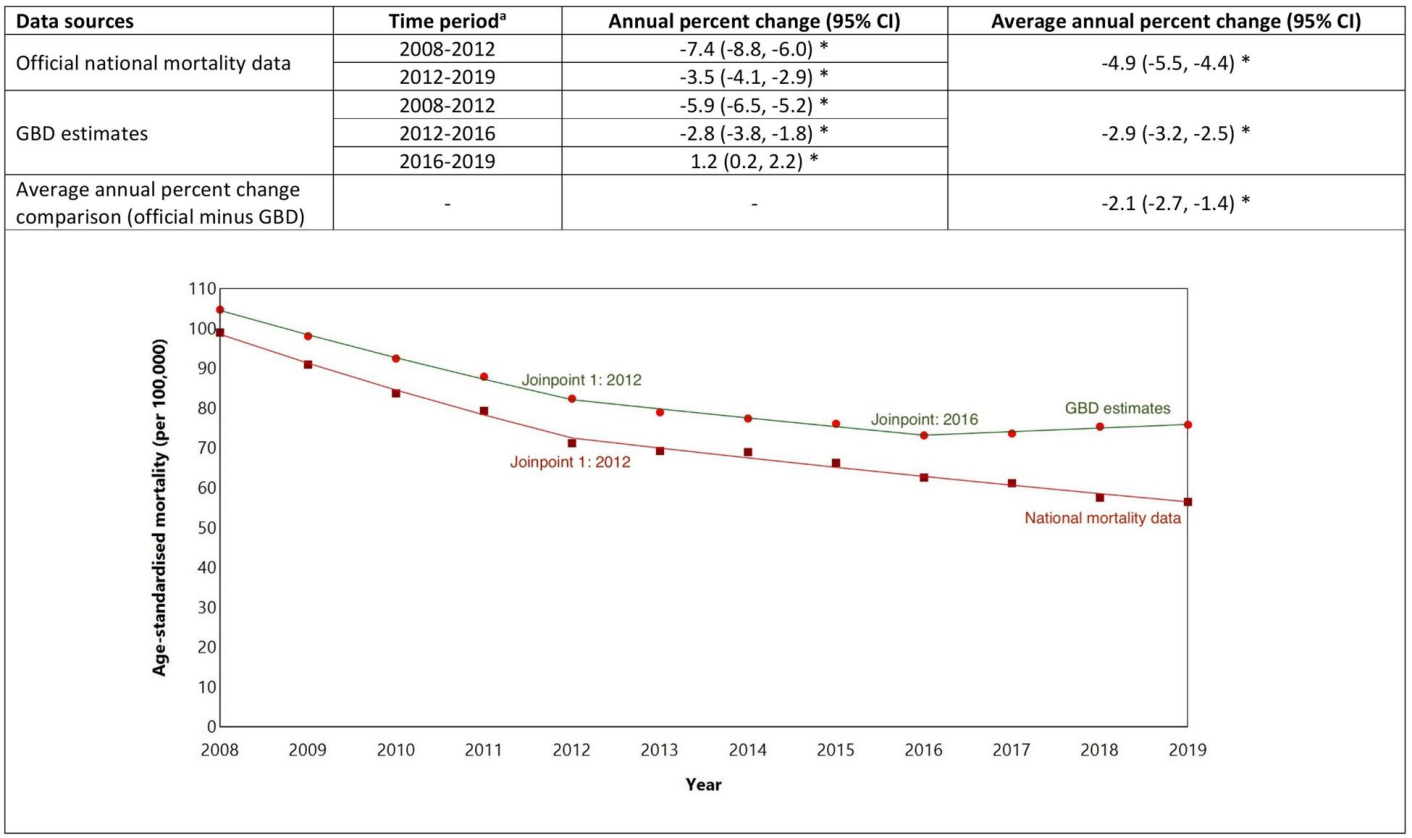
Trends in coronary heart disease mortality rates (2008–19) among the Australian population aged 35–84 years estimated using a Joinpoint model. ^a^Joinpoint regression identifies the points at which there is a statistically significant change in trends. ^b^An asterisk (*) indicates statistically significant difference from zero (alpha = 0.05 level).

Using official data, age-standardized mortality rates for CHD in Australia decreased from 99.0 per 100 000 people in 2008 to 56.5 per 100 000 in 2019 (Fig. 1); an average annual decrease of 4.9% (95% CI: 4.4%–5.5%) (Fig. 2). The Joinpoint analysis identified a statistically significant change in trends around 2012, with CHD mortality rates decreasing on average each year by 7.4% (6.0%–8.8%) from 2008 to 2012, and by 3.5% (2.9%–4.1%) between 2012 and 2019 (Fig. 2).

GBD estimates also showed an overall decrease in age-standardized CHD mortality rates from 2008 (104.7 per 100 000 people) to 2019 (75.8 per 100 000), with absolute rates considerably higher than those from national mortality data (Fig. 1). Over the study period, average annual CHD mortality decline was 2.9% (2.5%–3.2%) using GBD data, with estimates using official national mortality data being 2.1% (95% CI: 1.4%–2.7%) greater (Fig. 2). GBD estimates showed average annual decreases in CVD mortality of 5.9% (5.2%–6.5%) during 2008–12, and 2.8% (1.8%–3.8%) during 2012–16, with annual increases of 1.2% (0.2%–2.2%) during 2016–19 (Fig. 2 and Supplementary Table S2).

### Stroke mortality rates in Australia

**Figure 3.**
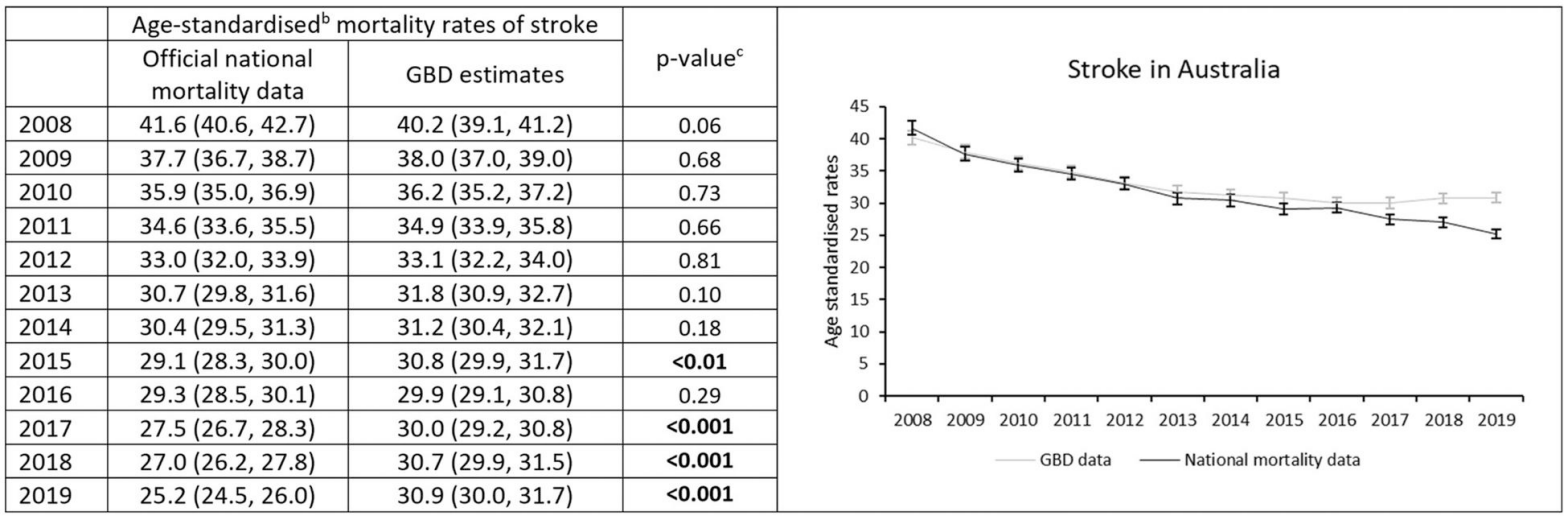
Stroke mortality rates from 2008 to 2019, estimated from official national data and the Global Burden of Disease study for the Australian population aged 35–84 years. ^a^Stroke defined according to International Classification of Disease codes of I60–I69, G45–G46. G codes are not included in the official national mortality data. ^b^Mortality rates are age-standardized to the Global Burden of Disease (GBD) Study World Standard Population. ^c^*P*-values were calculated using Wald test, with values less than 0.05 indicating a significant difference between the data sources.

**Figure 4.**
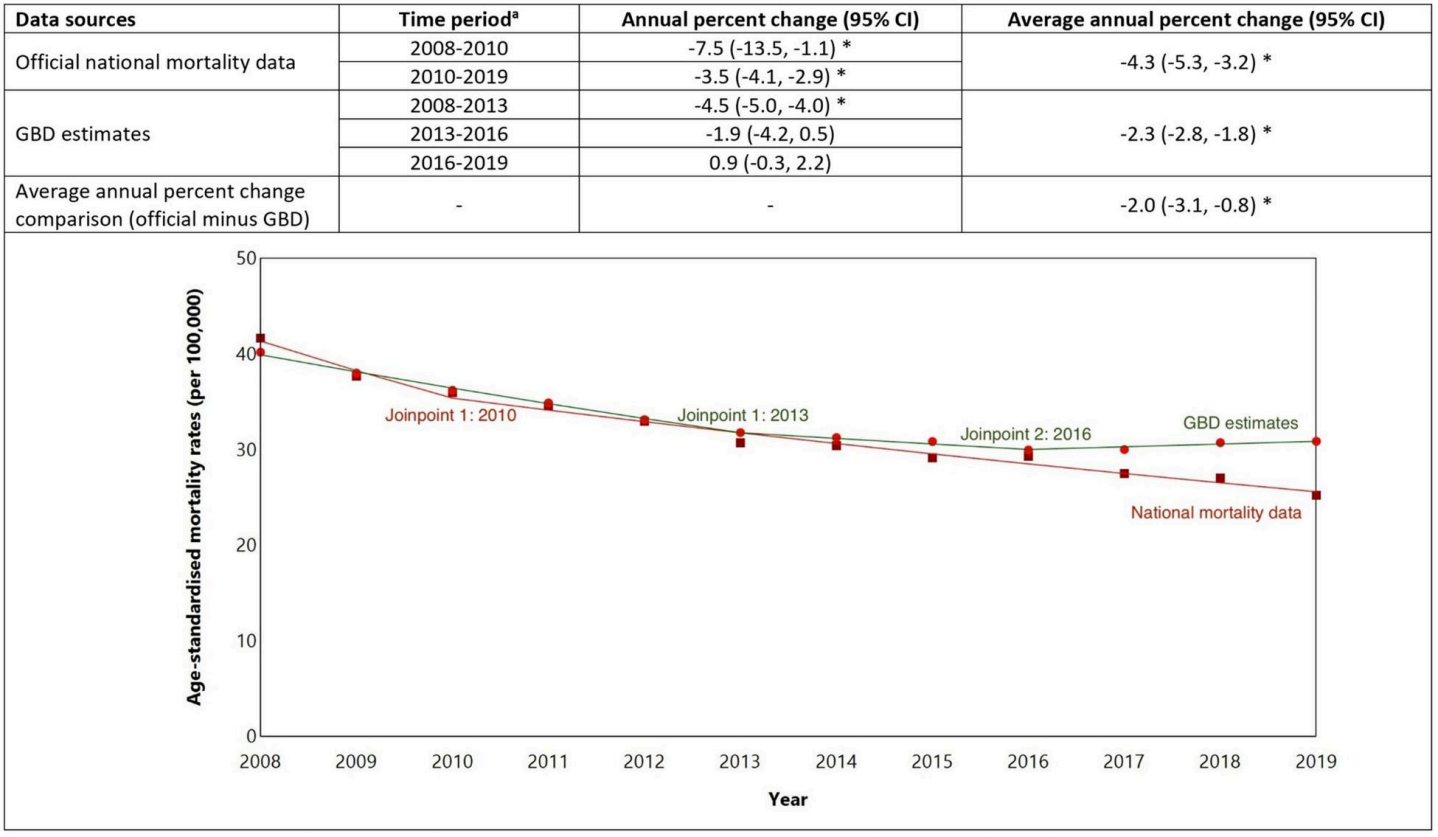
Trends in stroke mortality rates (2008–19) among the Australian population aged 35–84 years estimated using a Joinpoint model. ^a^Joinpoint regression identifies the points at which there is a statistically significant change in trends. ^b^An asterisk (*) indicates statistically significant difference from zero (alpha = 0.05 level).

Using official data, age-standardized stroke mortality rates declined from 41.6 per 100 000 people in 2008 to 25.2 per 100 000 in 2019 (Fig. 3), with average annual reductions of 4.3% (95% CI: 3.2%–5.3%) (Fig. 4). Joinpoint analysis showed stroke mortality decreased on average each year by 7.5% (1.1%–13.5%) during 2008–10, and 3.5% (2.9%–4.1%) during 2010–19 (Fig. 4).

GBD estimates also showed a decline in age-standardized stroke mortality rates from 2008 (40.2 per 100 000) to 2016 (29.9 per 100 000). However, after 2016, GBD stroke estimates were higher when compared to official national mortality data (e.g. 30.9 per 100 000 people versus 25.2 per 100 000; Fig. 3). Overall, from 2008 to 2019, GBD estimates showed an annual decline in stroke mortality of 2.3% (1.8%–2.8%); official national estimates showed an average 2.0% (0.8%–3.1%) greater annual percent decline in mortality than those from GBD (Fig. 4).

For GBD estimates, Joinpoint analysis results showed a 4.5% (95% CI: 4.0%–5.0%) average annual decline in stroke mortality during 2008–13, with no statistically significant changes between 2013–16 and 2016–19 (Fig. 4 and Supplementary Table S3).

### CHD mortality rates in New Zealand

**Figure 5.**
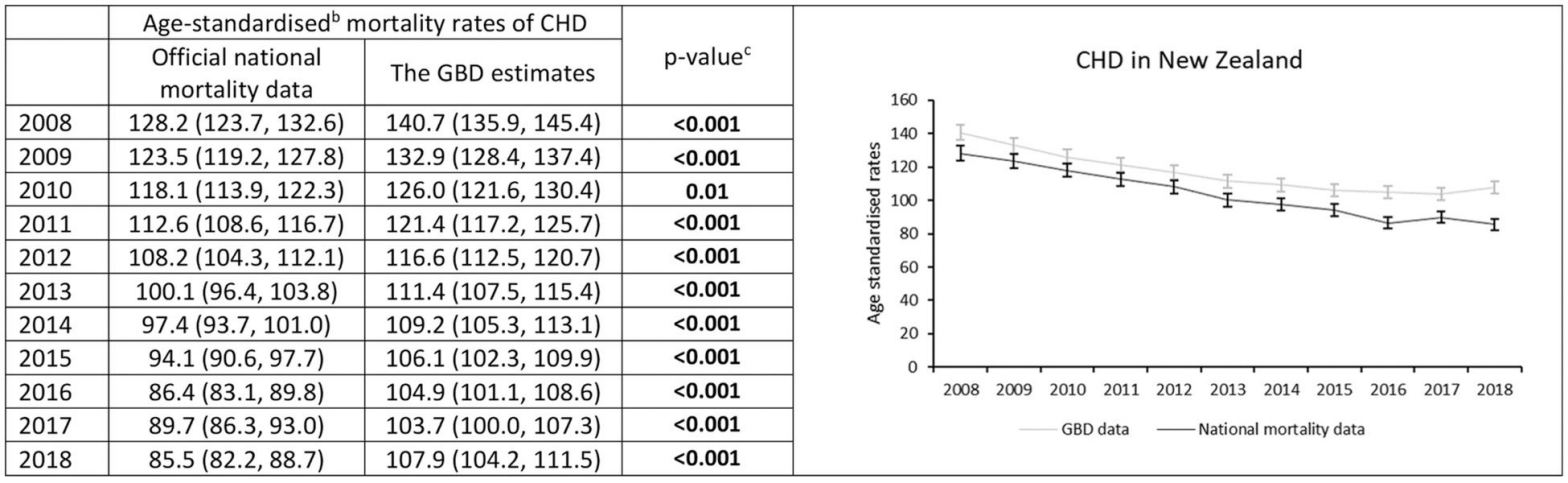
Coronary heart disease mortality rates from 2008 to 2018, estimated from official national data and the Global Burden of Disease study for the New Zealand population aged 35–84 years. ^a^Coronary heart disease (CHD) defined according to International Classification of Disease codes of I20–I25. ^b^Mortality rates are age-standardized to the Global Burden of Disease (GBD) Study World Standard Population. ^c^*P*-values were calculated using Wald test, with values less than 0.05 indicating a significant difference between the data sources.

**Figure 6.**
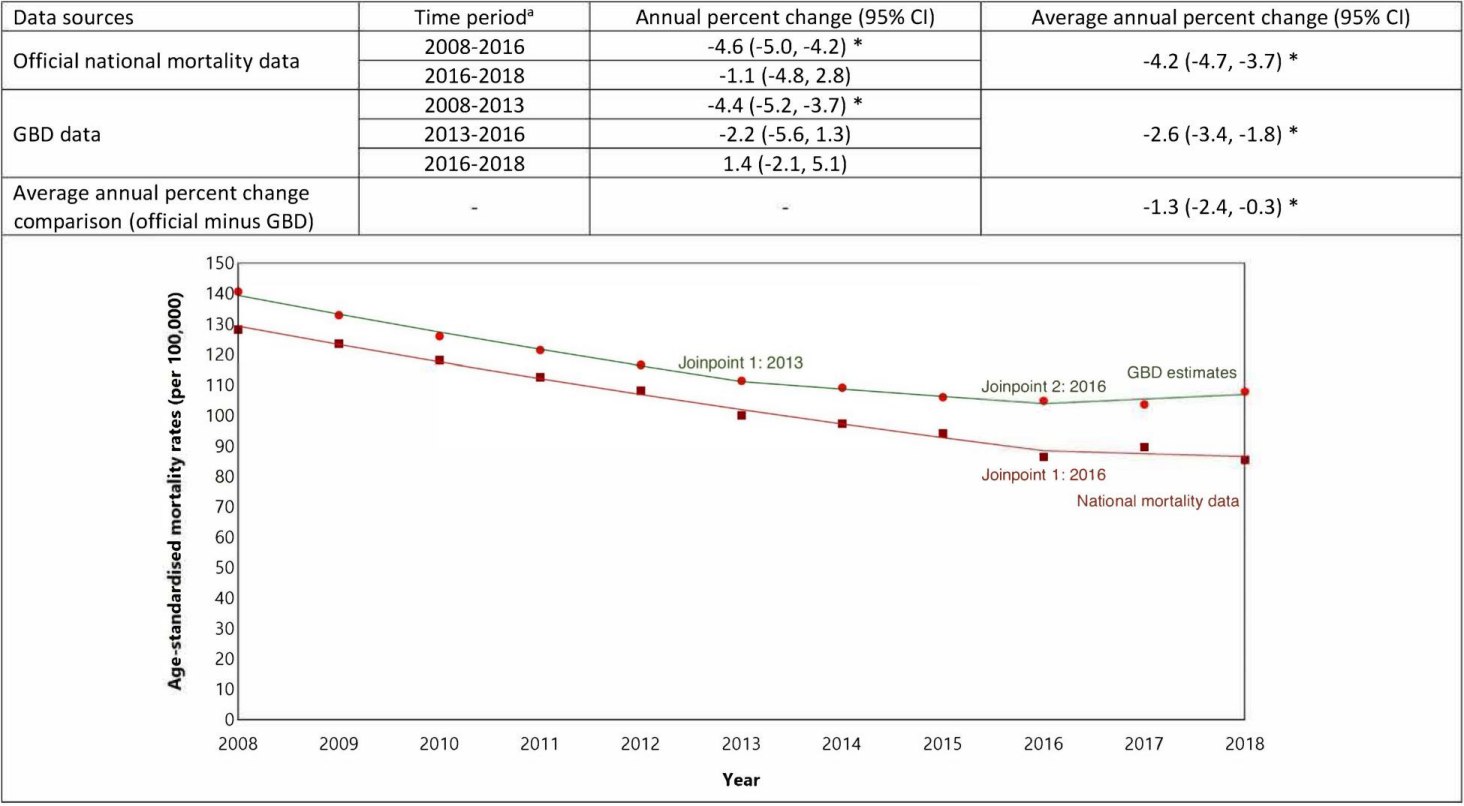
Trends in coronary heart disease mortality rates (2008–18) among the New Zealand population aged 35–84 years estimated using a Joinpoint model. ^a^Joinpoint regression identifies the points at which there is a statistically significant change in trends. ^b^An asterisk (*) indicates statistically significant difference from zero (alpha = 0.05 level).

Using official national New Zealand data, age-standardized CHD mortality rates decreased steadily from 128.2 per 100 000 people in 2008 to 85.5 per 100 000 in 2018 (Fig. 5). From 2008 to 2018 CHD mortality decreased on average by 4.2% (95% CI: 3.7%–4.7%) annually, with a significant change in the trend identified in 2016 (Fig. 6). Age-standardized CHD mortality rates declined by 4.6% (4.2%–5.0%) on average each year from 2008 to 2016 but did not change significantly between 2016 and 2018 (Fig. 6).

Although GBD estimates also showed a decline in age-standardized CHD mortality rates over the study period (140.7 per 100 000 in 2008 to 107.9 per 100 000 in 2018; Fig. 5), absolute rates were higher than those observed in official national mortality data. From 2008 to 2018, the average annual decline in CHD mortality using GBD estimates was 2.6% (1.8%–3.4%), meaning the official national statistics showed a 1.3% (0.3%–2.4%) greater annual decline in mortality than GBD (Fig. 6).

Between 2008 and 2013, age-standardized CHD mortality rates from GBD estimates declined by an average of 4.4% (3.7%–5.2%) but did not change significantly between 2013 and 2016 (average annual change −2.2% [−5.6% to 1.3%]) or between 2016 and 2018 (1.4% [−2.1% to 5.1%]; Fig. 6 and Supplementary Table S4), though there was substantial uncertainty in these recent estimates.

### Stroke mortality rates in New Zealand

**Figure 7.**
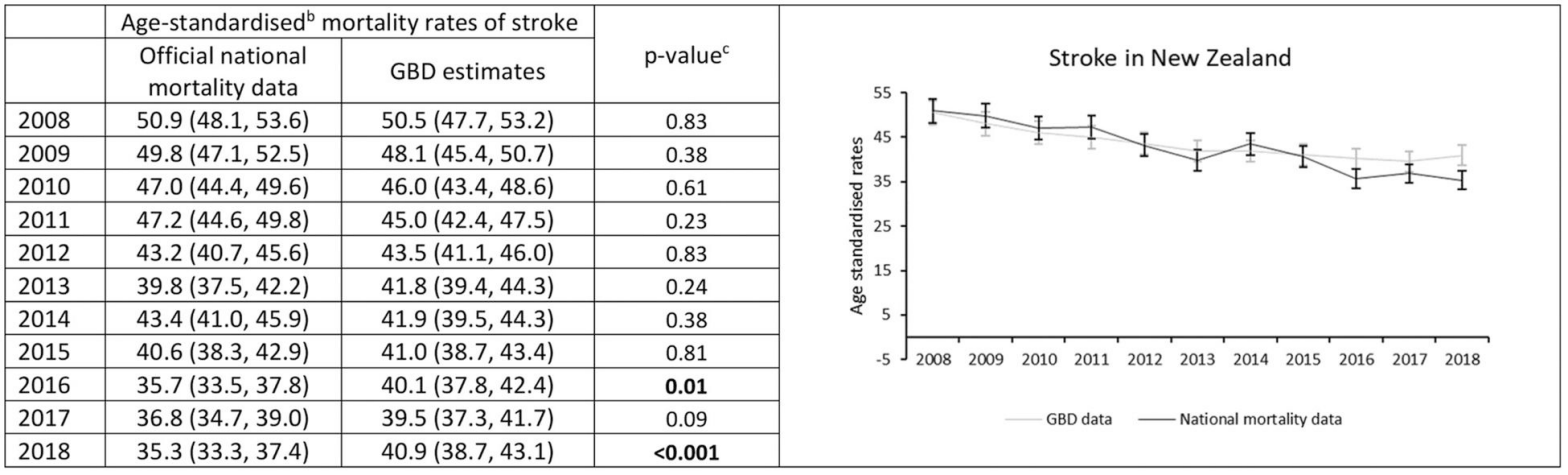
Stroke mortality rates from 2008 to 2018, estimated from official national data and the Global Burden of Disease study for the New Zealand population aged 35–84 years. ^a^Stroke defined according to International Classification of Disease codes of I60–I69, G45, and G46. ^b^Mortality rates are age-standardized to the Global Burden of Disease (GBD) Study World Standard Population. ^c^*P*-values were calculated using Wald test, with values less than 0.05 indicating a significant difference between the data sources.

**Figure 8.**
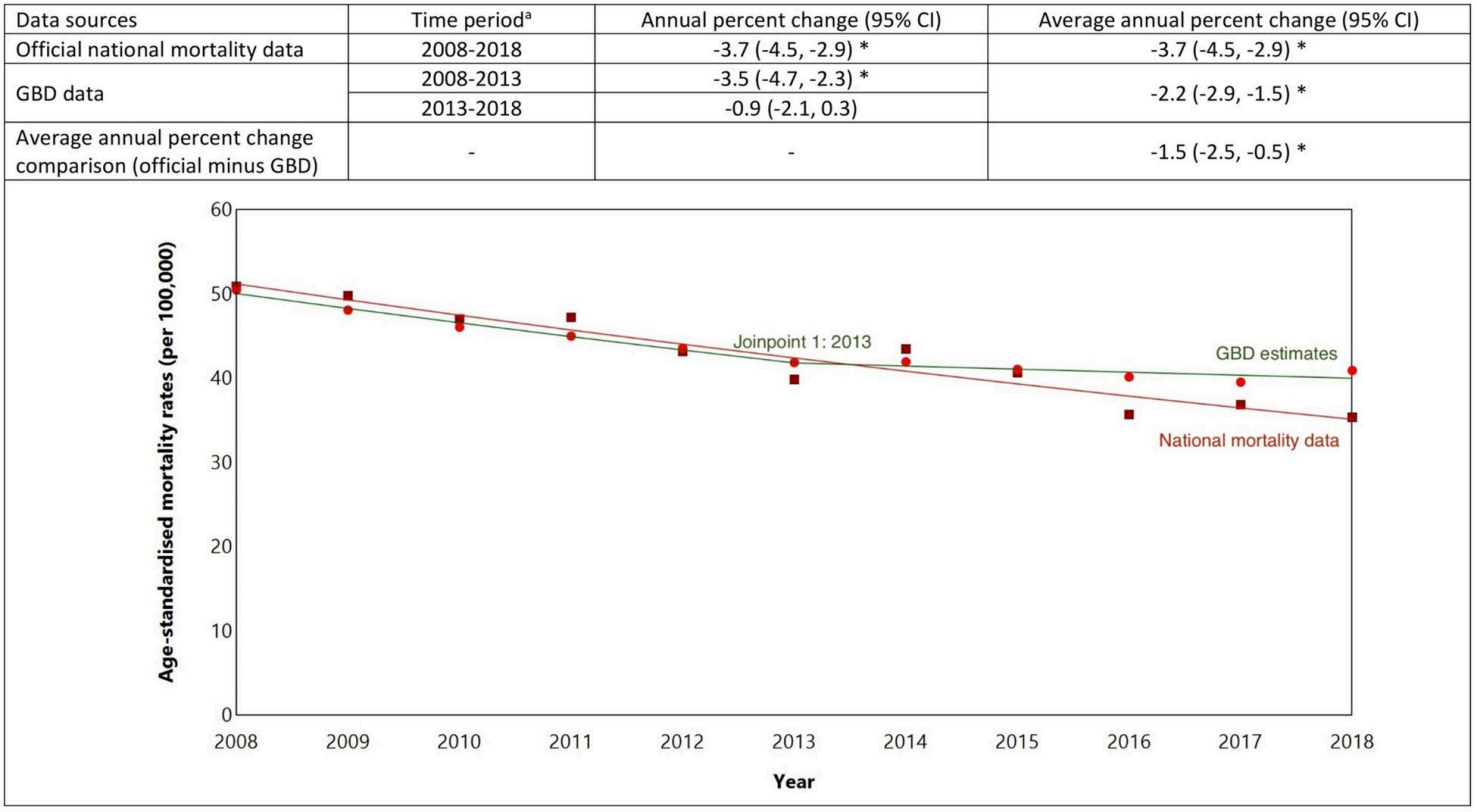
Trends in stroke mortality rates (2008–18) among the New Zealand population aged 35–84 years estimated using a Joinpoint model. ^a^Joinpoint regression identifies the points at which there is a statistically significant change in trends. ^b^An asterisk (*) indicates statistically significant difference from zero (alpha = 0.05 level).

Using official national data, age-standardized stroke mortality rates in New Zealand decreased from 50.9 per 100 000 people in 2008 to 35.3 per 100 000 in 2018 (Fig. 7), with an average annual decrease of 3.7% (95% CI: 2.9%–4.5%) (Fig. 8). No significant changes in the trend over time were identified.

GBD absolute stroke mortality rates were somewhat similar to those from the official national mortality data (Fig. 7), with GBD estimates showing a decline in age-standardized mortality rates from 50.5 per 100 000 people in 2008 to 40.9 per 100 000 in 2018; an average annual decline of 2.2% (1.5%–2.9%) (Fig. 8). Absolute stroke mortality rates using official national mortality data were 1.5% (0.5%–2.5%) greater, on average, compared to GBD estimates. However, in contrast to official national mortality estimates, GBD estimates showed that age-standardized stroke mortality rates for New Zealand appeared to stabilize between 2013 and 2018, with average annual declines of 3.5% (2.3%–4.7%) from 2008 to 2013 and no significant change from 2013 to 2018 (−0.9% [−2.1% to 0.3%]; Fig. 8 and Supplementary Table S5).

### Post-hoc analyses

Findings were similar when analysed separately for males and females (Supplementary Figs. S1–S8).

From 2008 to 2016, age-standardized mortality rates for all CVD for Australia were similar using official national mortality data and GBD estimates. However, from 2016 to 2019, CVD mortality rates diverged, with GBD estimates being substantially higher than official national data (Supplementary Fig. S9). In 2016 (the last year with observed data in GBD), GBD estimates were 3.2% higher than official national data (136.5 versus 132.3 per 100 000), widening to 17.6% higher (140.8 versus 119.7 per 100 000 people) in 2019 (Supplementary Fig. S9), suggesting out-of-sample predictions largely contributed to the observed differences.

## Discussion

Based on official national mortality data, age-standardized mortality rates for CHD and stroke showed a steady decline, with notable trend shifts. In Australia, CHD mortality fell by 7.4% annually on average from 2008 to 2012, then slowed to a 3.5% average annual decrease. Similarly, stroke mortality decreased on average by 7.5% annually from 2008 to 2010, and then by 3.5% annually on average thereafter. In New Zealand, CHD mortality declined annually by an average of 4.6% until 2016, then stabilized, while stroke mortality consistently decreased from 2008 to 2018.

There were discrepancies in absolute mortality rates and temporal trends when comparing official national mortality data and GBD estimates. First, GBD estimates showed an increase in CHD mortality in Australia from 2016 onwards and a stabilization of stroke mortality rates for both Australia and New Zealand for this same period. Although there was some evidence suggesting increasing CHD mortality rates in New Zealand between 2016 to 2018 based on GBD data, this did not achieve statistical significance. The differences between GBD and official data for the period 2016–2018/19 persisted in the post-hoc analysis of all CVD mortality rates (Supplementary Fig. S9) and is likely due to GBD estimates for Australia and New Zealand being based on projections from 2016. The evidence indicates that the assumptions underlying those projections led to trends differing from those observed in official national statistics.

Secondly, absolute CHD mortality rates in Australia and New Zealand were generally higher when calculated using GBD compared to official national data, while stroke mortality rates were somewhat similar, particularly from 2008 to 2016. Our results indicate this is likely due to the redistribution of ill-defined causes of death used by the GBD and might affect the CHD estimates more than the stroke estimates. Redistribution of causes of death is undertaken by GBD as some causes (frequently referred to as ‘garbage’ codes) are not considered informative for burden of disease estimations and are reallocated to other codes [24]. This is not a limitation in itself but should be considered when GBD statistics are being interpreted. In a post-hoc analysis using a composite outcome of all CVD—which should be less affected by distribution of ill-defined deaths—absolute CVD mortality rates from 2008 to 2014 for Australia were similar between official national mortality data and GBD estimates (Supplementary Fig. S9).

Our study provides updated estimates of CHD and stroke mortality trends for Australia and New Zealand and is the first to compare these to GBD estimates. Our findings of a slower but continuing decline in CHD and stroke mortality rates in more recent years are consistent with a previous report from AIHW that reported a slowing in CVD (including CHD and stroke) mortality rate declines over time in Australia up to 2017 [3]. Although the exact causes for this slowing are not known, it may be due to an increasing prevalence of some CVD risk factors. For example, the prevalence of diabetes in Australia almost doubled between 2000 and 2011/12 in Australia but has since stabilized [25]. Since there is a lag between risk factor exposure and CVD events, we would expect any changes in CVD mortality rates to be observed some years after changes in risk factors. Australia also has high and increasing prevalence of obesity, and previous analysis has shown that premature CVD mortality with co-morbidities related to obesity (e.g. diabetes, chronic kidney disease) increased in the mid-2010s [4]. Likewise, we may have yet to observe the full effects of declining tobacco smoking on CVD event and mortality rates [26]. We also found that absolute age-standardized CHD and stroke mortality rates were consistently higher in New Zealand than in Australia for every year in the study period, regardless of the data source (official mortality data or GBD data). This could be due to differences in risk factor prevalence, prevention approaches, and treatment between the two countries.

Our findings are also broadly consistent with a previous study that found GBD estimates were generally higher than those observed in the Australian Burden of Disease study for 47 out of 50 common diseases examined, with differences as large as >20% [27]. This previous study hypothesized that these differences were due to multiple factors, including disease classifications/definitions, underlying data sources, and redistribution of causes of death [27]. Our study provides novel evidence that differences in disease estimates from official national data and GBD estimates extend to CVD mortality data, that they impact on trend estimates, and that these differences are likely due to out-of-sample predictions, and to a smaller extent, redistribution of death.

GBD is designed to quantify the magnitude of major diseases, risk factors, and intermediate clinical outcomes in a highly standardized way, to allow for comparisons between countries, over time, and by age, and sex [28]. To enable comparison across regions, the GBD often uses modelling to process data [29, 30], and where national data are unavailable or unsuitable, projections are used to estimate future disease. Our results suggest that the finding of a recent increase in CHD and stroke mortality in Australian and New Zealand GBD estimates [15], based on its 2019 reports [8], is chiefly attributable to the use of projected mortality data from 2016.

Although we included 12 years of mortality data for Australia and 11 years for New Zealand, for some time periods in the Joinpoint regression, only 3 years of data were included, which may reduce statistical power. Although we used methods to enhance comparability between official national mortality rate and GBD estimates, there may be further differences that were not possible to account for. There is limited published information on exact data processing steps and how GBD generates mortality projections for years in which they have no access to mortality data. It was also not possible to replicate the GBD approach of redistribution of ill-defined causes of death as the exact algorithms used are not published. Due to data availability, our study focused on CVD mortality in Australia and New Zealand and was primarily restricted to data from before the COVID-19 pandemic. The effects of COVID-19 on overall CVD mortality rates in specific countries are unclear but may lead to changes in CVD death rates due to COVID-19 cardiovascular complications and reduced/delayed CVD treatment [31]. Future research is needed to assess whether there is evidence of similar differences between official mortality data and GBD data for other high-income countries.

Our findings have implications for the interpretation of estimates from the GBD, particularly where estimates are based on data projections. GBD estimates are calculated to provide the best possible comparison of disease estimates between regions, but caution should be applied when interpreting what these data mean for disease trends in individual countries and when considering trends that are estimated based on projected data. Transparency in GBD reporting also needs to be improved and publications should make prominent and clear which data are imputed or projected—including for specific years and countries—and provided greater information about processes used to transform data.

Findings from this and previous studies highlight the importance of basing disease mortality estimates on official national statistics and supporting the collection and generation of such statistics, rather than being inferred from GBD estimates, particularly when being used to evaluate or guide healthcare or policy decisions.

## Ethics approval

Ethics approval for this study was provided by the Aboriginal Health and Medical Research Council of NSW (reference: 1730/20).

## Supplementary Material

dyaf112_Supplementary_Data

## Data Availability

GBD estimates used in this article are publicly available from the GBD website, at https://vizhub.healthdata.org/gbd-results/. The official Australian national mortality data underlying this article are publicly available in GRIM books published by AIHW, at https://www.aihw.gov.au/reports/life-expectancy-deaths/grim-books/contents/grim-excel-workbooks. The official New Zealand national mortality data can be downloaded using the Mortality web tool at https://www.tewhatuora.govt.nz/our-health-system/data-and-statistics/mortality-web-tool/.
