# Supplementary material for: Coronary heart disease and stroke mortality trends in Australia and New Zealand: comparison of official national mortality data and Global Burden of Disease estimates
Source: Int J Epidemiol. 2025 Jul 2;54(4):dyaf112. doi: 10.1093/ije/dyaf112 (PMC12221867; doi:10.1093/ije/dyaf112)
Supplement: dyaf112_Supplementary_Data [file dyaf112_supplementary_data.docx]

**Coronary heart disease and stroke mortality trends in Australia and New Zealand: comparison of official national mortality data and Global Burden of Disease estimates**

Yuehan Zhang, Grace Joshy, Karen Bishop, Tim Adair, Wendy Ho, Katrina Sheehan, Michelle Gourley, Rod Jackson, Mai Nguyen, Emily Banks, Ellie Paige.

**Supplementary material**

**Contents**

[Figure S1: Coronary heart disease mortality rates from 2008 to 2019, estimated from official national mortality data and the Global Burden of Disease study for the Australian male population aged 35 to 84 years. 3](#_Toc199856874)

[Figure S2: Stroke mortality rates from 2008 to 2019, estimated from official national mortality data and the Global Burden of Disease study for the Australian male population aged 35 to 84 years. 4](#_Toc199856875)

[Figure S3: Coronary heart disease mortality rates from 2008 to 2018, estimated from official national mortality data and the Global Burden of Disease study for the New Zealand male population aged 35 to 84 years. 5](#_Toc199856876)

[Figure S4: Stroke mortality rates from 2008 to 2018, estimated from official national mortality data and the Global Burden of Disease study for the New Zealand male population aged 35 to 84 years. 6](#_Toc199856877)

[Figure S5: Coronary heart disease mortality rates from 2008 to 2019, estimated from official national mortality data and the Global Burden of Disease study for the Australian female population aged 35 to 84 years. 7](#_Toc199856878)

[Figure S6: Stroke mortality rates from 2008 to 2019, estimated from official national mortality data and the Global Burden of Disease study for the Australian female population aged 35 to 84 years. 8](#_Toc199856879)

[Figure S7: Coronary heart disease mortality rates from 2008 to 2018, estimated from official national mortality data and the Global Burden of Disease study for the New Zealand female population aged 35 to 84 years. 9](#_Toc199856880)

[Figure S8: Stroke mortality rates from 2008 to 2018, estimated from official national mortality data and the Global Burden of Disease study for the New Zealand female population aged 35 to 84 years. 10](#_Toc199856881)

[Figure S9: Cardiovascular disease mortality rates from 2008 to 2019, estimated from official national mortality data and the Global Burden of Disease study for the Australian population aged 35 to 84 years. 11](#_Toc199856882)

[Table S1: weights of different 5-year age groups of the 2019 standard population 12](#_Toc199856883)

[Table S2: Trends in coronary heart disease mortality rates (2008-2019) among the Australian population aged 35-84, estimated by Joinpoint regression model 13](#_Toc199856884)

[Table S3: Trends in stroke mortality rates (2008-2019) among the Australian population aged 35-84, estimated by Joinpoint regression model 14](#_Toc199856885)

[Table S4: Trends in coronary heart disease mortality rates (2008-2018) among the New Zealand population aged 35-84, estimated by Joinpoint regression model 15](#_Toc199856886)

[Table S5: Trends in stroke mortality rates (2008-2018) among the New Zealand population aged 35-84, estimated by Joinpoint regression model 16](#_Toc199856887)

**Supplementary analysis**

##### Figure S1: Coronary heart disease ^a^ mortality rates from 2008 to 2019, estimated from official national mortality data and the Global Burden of Disease study for the Australian male population aged 35 to 84 years.

|  | Age-standardised^b^ mortality rates of CHD | | p-value^c^ | 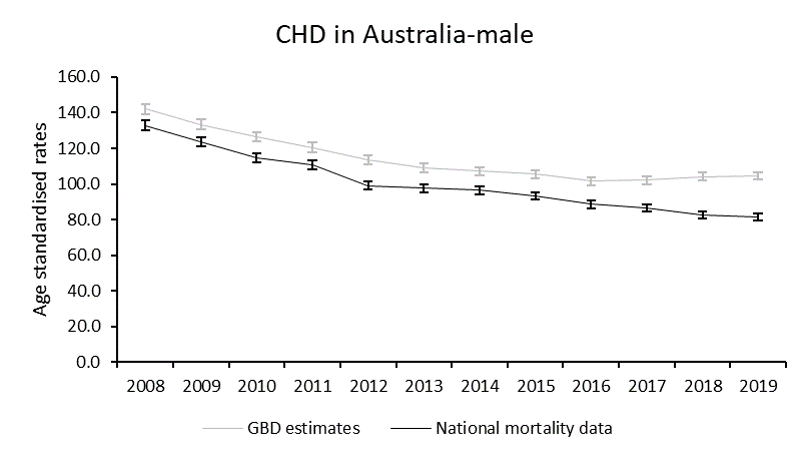 |
| --- | --- | --- | --- | --- |
|  | Official national mortality data | GBD estimates |  |  |
| 2008 | 132.8 (130.0, 135.6) | 142.0 (139.1, 144.9) | **<0.001** |  |
| 2009 | 123.7 (121.0, 126.4) | 133.3 (130.5, 136.1) | **<0.001** |  |
| 2010 | 114.4 (111.8, 117.0) | 126.5 (123.8, 129.2) | **<0.001** |  |
| 2011 | 110.5 (108.0, 113.0) | 120.5 (117.9, 123.1) | **<0.001** |  |
| 2012 | 99.2 (96.8, 101.5) | 113.3 (110.8, 115.8) | **<0.001** |  |
| 2013 | 97.6 (95.3, 99.9) | 109.0 (106.6, 111.5) | **<0.001** |  |
| 2014 | 96.5 (94.2, 98.7) | 107.1 (104.7, 109.5) | **<0.001** |  |
| 2015 | 93.2 (91.1, 95.4) | 105.5 (103.2, 107.9) | **<0.001** |  |
| 2016 | 88.5 (86.4, 90.6) | 101.5 (99.2, 103.8) | **<0.001** |  |
| 2017 | 86.3 (84.3, 88.4) | 102.2 (100.0, 104.5) | **<0.001** |  |
| 2018 | 82.6 (80.6, 84.6) | 104.0 (101.8, 106.2) | **<0.001** |  |
| 2019 | 81.4 (79.4, 83.3) | 104.5 (102.3, 106.7) | **<0.001** |  |

^a^Coronary heart disease (CHD) defined according to International Classification of Disease codes of I20-I25

^b^Mortality rates are age-standardised to the Global Burden of Disease Study World Standard Population

^c^p-values were calculated using Wald test, with values less than 0.05 indicating a significant difference between the data sources.

##### Figure S2: Stroke^a^ mortality rates from 2008 to 2019, estimated from official national mortality data and the Global Burden of Disease study for the Australian male population aged 35 to 84 years.

|  | Age-standardised^b^ mortality rates of stroke | | p-value^c^ | 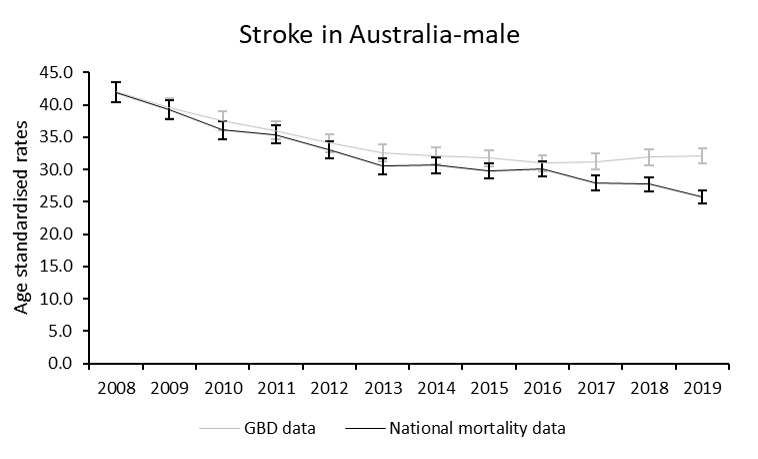 |
| --- | --- | --- | --- | --- |
|  | Official national mortality data | GBD estimates |  |  |
| 2008 | 41.9 (40.4, 43.5) | 42.0 (40.5, 43.6) | 0.92 |  |
| 2009 | 39.3 (37.8, 40.7) | 39.5 (38.0, 41.0) | 0.82 |  |
| 2010 | 36.1 (34.7, 37.5) | 37.6 (36.1, 39.0) | 0.15 |  |
| 2011 | 35.4 (34.0, 36.8) | 36.0 (34.6, 37.4) | 0.55 |  |
| 2012 | 33.0 (31.7, 34.3) | 34.1 (32.7, 35.4) | 0.26 |  |
| 2013 | 30.5 (29.3, 31.8) | 32.6 (31.3, 33.9) | **0.03** |  |
| 2014 | 30.7 (29.5, 32.0) | 32.1 (30.8, 33.4) | 0.13 |  |
| 2015 | 29.8 (28.6, 31.0) | 31.8 (30.5, 33.0) | **0.02** |  |
| 2016 | 30.1 (28.9, 31.3) | 31.0 (29.8, 32.2) | 0.30 |  |
| 2017 | 28.0 (26.8, 29.1) | 31.3 (30.1, 32.5) | **<0.001** |  |
| 2018 | 27.7 (26.6, 28.9) | 31.9 (30.7, 33.1) | **<0.001** |  |
| 2019 | 25.8 (24.7, 26.8) | 32.1 (31.0, 33.3) | **<0.001** |  |

^a^Stroke defined according to International Classification of Disease codes of I60-I69, G45 and G46. G codes are not included in the official national mortality data

^b^Mortality rates are age-standardised to the Global Burden of Disease Study World Standard Population

^c^p-values were calculated using Wald test, with values less than 0.05 indicating a significant difference between the data sources

##### Figure S3: Coronary heart disease^a^ mortality rates from 2008 to 2018, estimated from official national mortality data and the Global Burden of Disease study for the New Zealand male population aged 35 to 84 years.

|  | Age-standardised^b^ mortality rates of CHD | | p-value^c^ | 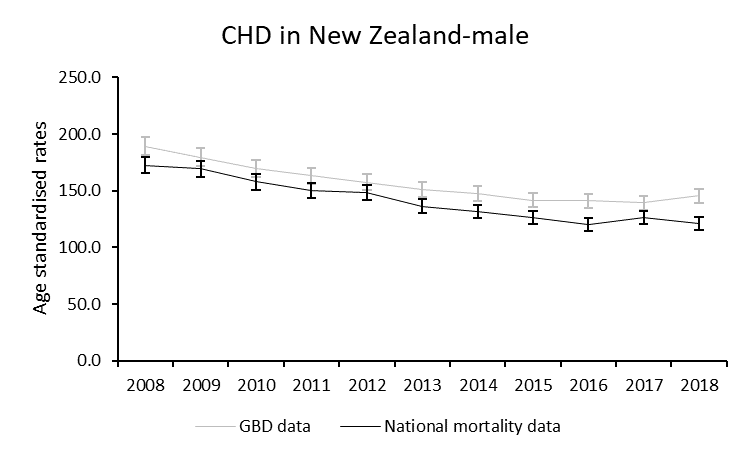 |
| --- | --- | --- | --- | --- |
|  | Official national mortality data | The GBD estimates |  |  |
| 2008 | 172.6 (165.2, 180.0) | 189.4 (181.5, 197.2) | **0.002** |  |
| 2009 | 169.3 (162.0, 176.5) | 179.7 (172.2, 187.3) | >0.05 |  |
| 2010 | 158.0 (151.1, 164.8) | 169.6 (162.4, 176.9) | **0.02** |  |
| 2011 | 150.3 (143.6, 156.9) | 163.1 (156.1, 170.1) | **0.01** |  |
| 2012 | 148.6 (142.1, 155.1) | 157.5 (150.7, 164.3) | 0.06 |  |
| 2013 | 136.2 (130.0, 142.4) | 151.1 (144.5, 157.7) | **0.001** |  |
| 2014 | 131.8 (125.8, 137.8) | 147.5 (141.0, 153.9) | **<0.001** |  |
| 2015 | 126.3 (120.5, 132.1) | 141.7 (135.5, 147.9) | **<0.001** |  |
| 2016 | 120.0 (114.4, 125.6) | 141.0 (134.9, 147.1) | **<0.001** |  |
| 2017 | 126.4 (120.7, 132.0) | 139.3 (133.3, 145.3) | **0.002** |  |
| 2018 | 121.0 (115.5, 126.4) | 145.6 (139.5, 151.7) | **<0.001** |  |

^a^Coronary heart disease (CHD) defined according to International Classification of Disease codes of I20-I25

^b^Mortality rates are age-standardised to the Global Burden of Disease Study World Standard Population

^c^p-values were calculated using Wald test, with values less than 0.05 indicating a significant difference between the data sources.

##### Figure S4: Stroke^a^ mortality rates from 2008 to 2018, estimated from official national mortality data and the Global Burden of Disease study for the New Zealand male population aged 35 to 84 years.

|  | Age-standardised^b^ mortality rates of stroke | | p-value^c^ | 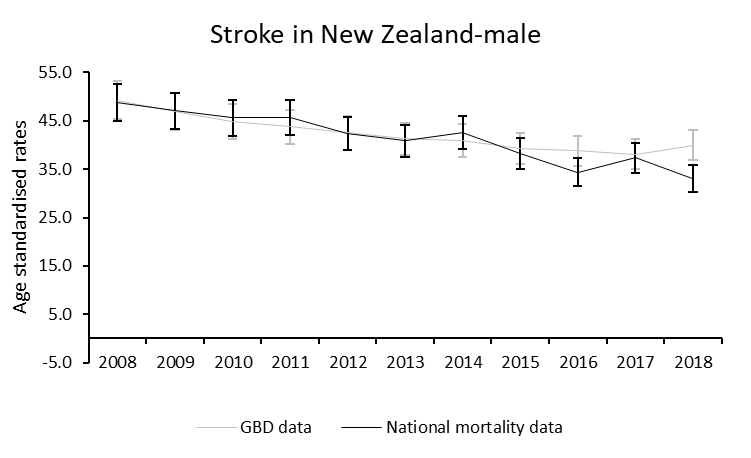 |
| --- | --- | --- | --- | --- |
|  | Official national mortality data | GBD estimates |  |  |
| 2008 | 48.7 (44.9, 52.6) | 49.2 (45.3, 53.1) | 0.86 |  |
| 2009 | 47.1 (43.3, 50.8) | 47.0 (43.2, 50.7) | 0.97 |  |
| 2010 | 45.6 (41.9, 49.2) | 44.8 (41.2, 48.5) | 0.77 |  |
| 2011 | 45.7 (42.1, 49.3) | 43.7 (40.2, 47.3) | 0.45 |  |
| 2012 | 42.4 (38.9, 45.8) | 42.5 (39.1, 46.0) | 0.94 |  |
| 2013 | 40.9 (37.5, 44.2) | 41.2 (37.9, 44.6) | 0.88 |  |
| 2014 | 42.7 (39.3, 46.0) | 40.9 (37.6, 44.2) | 0.47 |  |
| 2015 | 38.2 (35.1, 41.4) | 39.2 (36.0, 42.5) | 0.66 |  |
| 2016 | 34.4 (31.4, 37.3) | 38.8 (35.6, 41.9) | **0.05** |  |
| 2017 | 37.3 (34.3, 40.4) | 38.1 (35.1, 41.2) | 0.72 |  |
| 2018 | 33.1 (30.3, 35.9) | 39.9 (36.8, 43.0) | **0.001** |  |

^a^Stroke defined according to International Classification of Disease codes of I60-I69, G45 and G46.

^b^Mortality rates are age-standardised to the Global Burden of Disease Study World Standard Population

^c^p-values were calculated using Wald test, with values less than 0.05 indicating a significant difference between the data sources.

##### Figure S5: Coronary heart disease^a^ mortality rates from 2008 to 2019, estimated from official national mortality data and the Global Burden of Disease study for the Australian female population aged 35 to 84 years.

|  | Age-standardised^b^ mortality rates of CHD | | p-value^c^ | 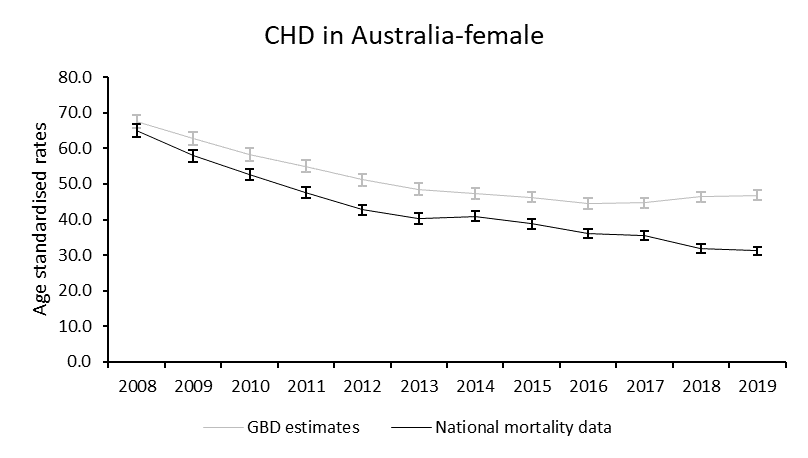 |
| --- | --- | --- | --- | --- |
|  | Official national mortality data | GBD estimates |  |  |
| 2008 | 65.0 (63.1, 66.9) | 67.5 (65.6, 69.5) | 0.07 |  |
| 2009 | 57.9 (56.1, 59.7) | 62.8 (61.0, 64.7) | **<0.001** |  |
| 2010 | 52.6 (51.0, 54.3) | 58.2 (56.4, 60.0) | **<0.001** |  |
| 2011 | 47.7 (46.1, 49.3) | 55.0 (53.3, 56.7) | **<0.001** |  |
| 2012 | 42.7 (41.2, 44.2) | 51.1 (49.5, 52.8) | **<0.001** |  |
| 2013 | 40.3 (38.9, 41.8) | 48.5 (47.0, 50.1) | **<0.001** |  |
| 2014 | 40.9 (39.5, 42.4) | 47.4 (45.8, 48.9) | **<0.001** |  |
| 2015 | 38.8 (37.4, 40.2) | 46.3 (44.8, 47.8) | **<0.001** |  |
| 2016 | 36.1 (34.8, 37.4) | 44.5 (43.0, 45.9) | **<0.001** |  |
| 2017 | 35.5 (34.2, 36.8) | 44.7 (43.3, 46.2) | **<0.001** |  |
| 2018 | 31.9 (30.7, 33.2) | 46.4 (44.9, 47.8) | **<0.001** |  |
| 2019 | 31.2 (30.0, 32.3) | 46.8 (45.4, 48.2) | **<0.001** |  |

^a^Coronary heart disease (CHD) defined according to International Classification of Disease codes of I20-I25

^b^Mortality rates are age-standardised to the Global Burden of Disease Study World Standard Population

^c^p-values were calculated using Wald test, with values less than 0.05 indicating a significant difference between the data sources.

##### Figure S6: Stroke^a^ mortality rates from 2008 to 2019, estimated from official national mortality data and the Global Burden of Disease study for the Australian female population aged 35 to 84 years.

|  | Age-standardised^b^ mortality rates of stroke | | p-value^c^ | 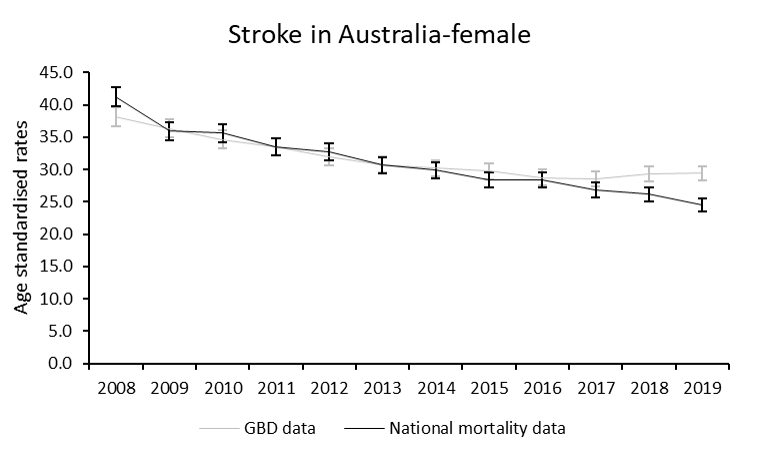 |
| --- | --- | --- | --- | --- |
|  | Official National mortality data | GBD estimates |  |  |
| 2008 | 52.9 (48.9, 56.8) | 51.6 (47.6, 55.5) | **0.004** |  |
| 2009 | 52.3 (48.4, 56.2) | 49.0 (45.2, 52.8) | 0.72 |  |
| 2010 | 48.3 (44.5, 52.0) | 47.1 (43.4, 50.8) | 0.32 |  |
| 2011 | 48.6 (44.9, 52.3) | 46.1 (42.5, 49.7) | 0.98 |  |
| 2012 | 43.9 (40.4, 47.3) | 44.4 (40.9, 48.0) | 0.41 |  |
| 2013 | 38.8 (35.5, 42.0) | 42.3 (38.9, 45.8) | 0.94 |  |
| 2014 | 44.2 (40.8, 47.7) | 42.8 (39.4, 46.2) | 0.73 |  |
| 2015 | 42.9 (39.6, 46.3) | 42.8 (39.4, 46.1) | 0.11 |  |
| 2016 | 36.9 (33.8, 39.9) | 41.4 (38.1, 44.7) | 0.63 |  |
| 2017 | 36.2 (33.2, 39.2) | 40.8 (37.6, 44.1) | **0.04** |  |
| 2018 | 37.4 (34.4, 40.5) | 41.8 (38.6, 45.0) | **<0.0001** |  |
| 2019 | 52.9 (48.9, 56.8) | 51.6 (47.6, 55.5) | **<0.0001** |  |

^a^Stroke defined according to International Classification of Disease codes of I60-I69, G45 and G46. G codes are not included in the official national mortality data

^b^Mortality rates are age-standardised to the Global Burden of Disease Study World Standard Population

^c^p-values were calculated using Wald test, with values less than 0.05 indicating a significant difference between the data sources.

##### Figure S7: Coronary heart disease^a^ mortality rates from 2008 to 2018, estimated from official national mortality data and the Global Burden of Disease study for the New Zealand female population aged 35 to 84 years.

|  | Age-standardised^b^ mortality rates of CHD | | p-value^c^ | 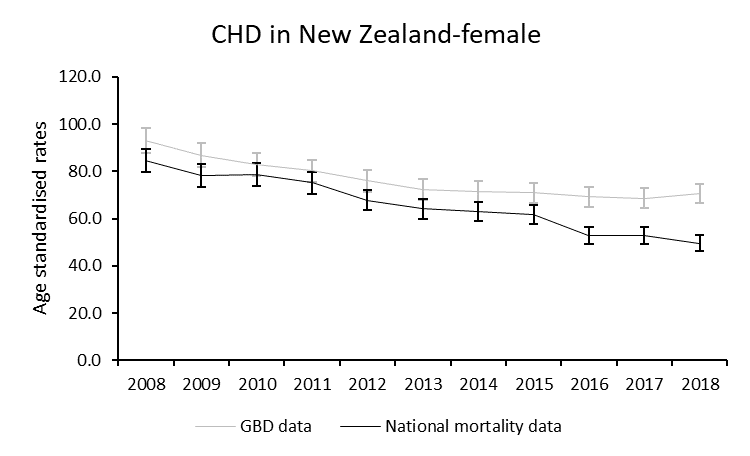 |
| --- | --- | --- | --- | --- |
|  | Official national mortality data | The GBD estimates |  |  |
| 2008 | 84.5 (79.5, 89.5) | 92.9 (87.6, 98.2) | 0.02 |  |
| 2009 | 78.4 (73.6, 83.2) | 86.8 (81.8, 91.9) | 0.02 |  |
| 2010 | 78.7 (73.9, 83.4) | 83.0 (78.1, 87.9) | 0.21 |  |
| 2011 | 75.2 (70.6, 79.8) | 80.2 (75.4, 85.0) | 0.15 |  |
| 2012 | 67.8 (63.5, 72.2) | 76.0 (71.4, 80.7) | 0.01 |  |
| 2013 | 64.2 (60.0, 68.3) | 72.2 (67.7, 76.6) | 0.01 |  |
| 2014 | 63.0 (58.9, 67.1) | 71.3 (67.0, 75.7) | 0.01 |  |
| 2015 | 62.0 (57.9, 66.0) | 70.9 (66.6, 75.2) | **0.003** |  |
| 2016 | 52.8 (49.1, 56.5) | 69.3 (65.0, 73.5) | **<0.001** |  |
| 2017 | 52.8 (49.2, 56.5) | 68.7 (64.5, 72.8) | **<0.001** |  |
| 2018 | 49.6 (46.2, 53.1) | 70.7 (66.6, 74.9) | **<0.001** |  |

^a^Coronary heart disease (CHD) defined according to International Classification of Disease codes of I20-I25

^b^Mortality rates are age-standardised to the Global Burden of Disease Study World Standard Population

^c^p-values were calculated using Wald test, with values less than 0.05 indicating a significant difference between the data sources.

##### Figure S8: Stroke^a^ mortality rates from 2008 to 2018, estimated from official national mortality data and the Global Burden of Disease study for the New Zealand female population aged 35 to 84 years.

|  | Age-standardised^b^ mortality rates of stroke | | p-value^c^ | 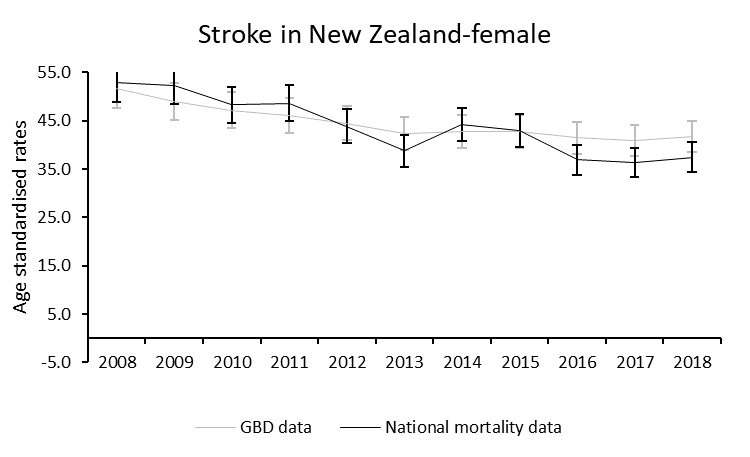 |
| --- | --- | --- | --- | --- |
|  | Official national mortality data | GBD estimates |  |  |
| 2008 | 52.9 (48.9, 56.8) | 51.6 (47.6, 55.5) | 0.64 |  |
| 2009 | 52.3 (48.4, 56.2) | 49.0 (45.2, 52.8) | 0.24 |  |
| 2010 | 48.3 (44.5, 52.0) | 47.1 (43.4, 50.8) | 0.68 |  |
| 2011 | 48.6 (44.9, 52.3) | 46.1 (42.5, 49.7) | 0.34 |  |
| 2012 | 43.9 (40.4, 47.3) | 44.4 (40.9, 48.0) | 0.82 |  |
| 2013 | 38.8 (35.5, 42.0) | 42.3 (38.9, 45.8) | 0.14 |  |
| 2014 | 44.2 (40.8, 47.7) | 42.8 (39.4, 46.2) | 0.57 |  |
| 2015 | 42.9 (39.6, 46.3) | 42.8 (39.4, 46.1) | 0.94 |  |
| 2016 | 36.9 (33.8, 39.9) | 41.4 (38.1, 44.7) | **0.05** |  |
| 2017 | 36.2 (33.2, 39.2) | 40.8 (37.6, 44.1) | **0.04** |  |
| 2018 | 37.4 (34.4, 40.5) | 41.8 (38.6, 45.0) | >0.05 |  |

^a^Stroke defined according to International Classification of Disease codes of I60-I69, G45 and G46.

^b^Mortality rates are age-standardised to the Global Burden of Disease Study World Standard Population

^c^p-values were calculated using Wald test, with values less than 0.05 indicating a significant difference between the data sources.

##### Figure S9: Cardiovascular disease^a^ mortality rates from 2008 to 2019, estimated from official national mortality data and the Global Burden of Disease study for the Australian population aged 35 to 84 years.

|  | Age-standardised^b^ mortality rates of composite CVD outcome | | p-value^c^ | 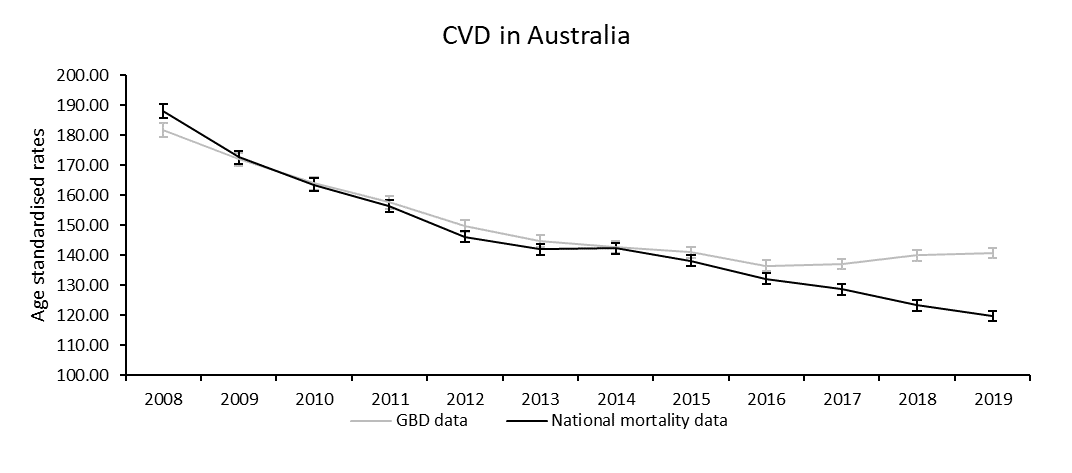 |
| --- | --- | --- | --- | --- |
|  | Official national mortality data | GBD estimates |  |  |
| 2008 | 188.0 (185.7, 190.4) | 181.7 (179.4, 184.0) | **<0.001** |  |
| 2009 | 172.6 (170.4, 174.8) | 172.0 (169.8, 174.2) | 0.59 |  |
| 2010 | 163.5 (161.4, 165.6) | 164.0 (161.9, 166.2) | 0.74 |  |
| 2011 | 156.5 (154.4, 158.6) | 157.7 (155.6, 159.7) | 0.42 |  |
| 2012 | 146.3 (144.3, 148.2) | 149.8 (147.8, 151.8) | **0.01** |  |
| 2013 | 142.0 (140.1, 143.9) | 144.8 (142.9, 146.8) | **0.04** |  |
| 2014 | 142.3 (140.4, 144.2) | 142.8 (140.8, 144.7) | 0.72 |  |
| 2015 | 138.2 (136.3, 140.0) | 141.0 (139.1, 142.9) | **0.04** |  |
| 2016 | 132.3 (130.5, 134.1) | 136.5 (134.7, 138.4) | **0.001** |  |
| 2017 | 128.7 (126.9, 130.5) | 137.1 (135.3, 138.9) | **<0.001** |  |
| 2018 | 123.3 (121.6, 125.0) | 140.1 (138.3, 141.9) | **<0.001** |  |
| 2019 | 119.7 (118.0, 121.3) | 140.8 (139.0, 142.5) | **<0.001** |  |

^a^Cardiovascular disease (CVD) defined according to International Classification of Disease codes of I00-I99

^b^Mortality rates are age-standardised to the Global Burden of Disease Study World Standard Population

^c^p-values were calculated using Wald test, with values less than 0.05 indicating a significant difference between the data sources

##### Table S1: weights of different 5-year age groups of the 2019 standard population

| 35 Years - 39 Years | 16.46% |
| --- | --- |
| 40 Years - 44 Years | 15.02% |
| 45 Years - 49 Years | 14.42% |
| 50 Years - 54 Years | 13.29% |
| 55 Years - 59 Years | 11.29% |
| 60 Years - 64 Years | 9.51% |
| 65 Years - 69 Years | 7.87% |
| 70 Years - 74 Years | 5.69% |
| 75 Years - 79 Years | 3.87% |
| 80 Years - 84 Years | 2.57% |

##### Table S2: Trends in coronary heart disease mortality rates (2008-2019) among the Australian population aged 35-84, estimated by Joinpoint regression model

|  | **Official national mortality data** | | | **GBD estimates** | | |
| --- | --- | --- | --- | --- | --- | --- |
| **Year** | **Age-standardised mortality rates** | **Joinpoint location** | **Annual Percent Change** | **Age-standardised mortality rates** | **Joinpoint location** | **Annual Percent Change** |
| 2008 | 99.0 (97.3, 100.7) |  | -7.4 (-8.8, -6.0)* | 104.7 (103.0, 106.5) |  | -5.9 (-6.5, -5.2)* |
| 2009 | 90.9 (89.3, 92.6) |  | -7.4 (-8.8, -6.0)* | 98.1 (96.4, 99.8) |  | -5.9 (-6.5, -5.2)* |
| 2010 | 83.7 (82.2, 85.2) |  | -7.4 (-8.8, -6.0)* | 92.4 (90.8, 94.1) |  | -5.9 (-6.5, -5.2)* |
| 2011 | 79.3 (77.9, 80.8) |  | -7.4 (-8.8, -6.0)* | 87.9 (86.3, 89.4) |  | -5.9 (-6.5, -5.2)* |
| 2012 | 71.2 (69.8, 72.6) | Joinpoint 1 |  | 82.4 (80.9, 83.9) | Joinpoint 1 |  |
| 2013 | 69.3 (67.9, 70.6) |  | -3.5 (-4.1, -2.9)* | 79.0 (77.5, 80.4) |  | -2.8 (-3.8, -1.8)* |
| 2014 | 69.0 (67.6, 70.3) |  | -3.5 (-4.1, -2.9)* | 77.4 (76.0, 78.8) |  | -2.8 (-3.8, -1.8)* |
| 2015 | 66.3 (65.0, 67.6) |  | -3.5 (-4.1, -2.9)* | 76.1 (74.7, 77.5) |  | -2.8 (-3.8, -1.8)* |
| 2016 | 62.6 (61.3, 63.8) |  | -3.5 (-4.1, -2.9)* | 73.1 (71.8, 74.5) | Joinpoint 2 |  |
| 2017 | 61.2 (59.9, 62.4) |  | -3.5 (-4.1, -2.9)* | 73.7 (72.3, 75.0) |  | 1.2 (0.2, 2.2)* |
| 2018 | 57.5 (56.4, 58.7) |  | -3.5 (-4.1, -2.9)* | 75.4 (74.0, 76.7) |  | 1.2 (0.2, 2.2)* |
| 2019 | 56.5 (55.3, 57.6) |  | -3.5 (-4.1, -2.9)* | 75.8 (74.5, 77.1) |  | 1.2 (0.2, 2.2)* |

***Indicates that the Annual Percent Change is significantly different from zero at the alpha = 0.05 level.**

##### Table S3: Trends in stroke mortality rates (2008-2019) among the Australian population aged 35-84, estimated by Joinpoint regression model

|  | **Official national mortality data** | | | **GBD estimates** | | |
| --- | --- | --- | --- | --- | --- | --- |
| **Year** | **Age-standardised mortality rates** | **Joinpoint location** | **Annual Percent Change** | **Age-standardised mortality rates** | **Joinpoint location** | **Annual Percent Change** |
| 2008 | 41.6 (40.6, 42.7) |  | -7.5 (-13.5, -1.1)* | 40.2 (39.1, 41.2) |  | -4.5 (-5.0, -4.0)* |
| 2009 | 37.7 (36.7, 38.7) |  | -7.5 (-13.5, -1.1)* | 38.0 (37.0, 39.0) |  | -4.5 (-5.0, -4.0)* |
| 2010 | 35.9 (35.0, 36.9) | Joinpoint 1 |  | 36.2 (35.2, 37.2) |  | -4.5 (-5.0, -4.0)* |
| 2011 | 34.6 (33.6, 35.5) |  | -3.5 (-4.1, -2.9)* | 34.9 (33.9, 35.8) |  | -4.5 (-5.0, -4.0)* |
| 2012 | 33.0 (32.0, 33.9) |  | -3.5 (-4.1, -2.9)* | 33.1 (32.2, 34.0) |  | -4.5 (-5.0, -4.0)* |
| 2013 | 30.7 (29.8, 31.6) |  | -3.5 (-4.1, -2.9)* | 31.8 (30.9, 32.7) | Joinpoint 1 |  |
| 2014 | 30.4 (29.5, 31.3) |  | -3.5 (-4.1, -2.9)* | 31.2 (30.4, 32.1) |  | -1.9 (-4.2, 0.5) |
| 2015 | 29.1 (28.3, 30.0) |  | -3.5 (-4.1, -2.9)* | 30.8 (29.9, 31.7) |  | -1.9 (-4.2, 0.5) |
| 2016 | 29.3 (28.5, 30.1) |  | -3.5 (-4.1, -2.9)* | 29.9 (29.1, 30.8) | Joinpoint 2 |  |
| 2017 | 27.5 (26.7, 28.3) |  | -3.5 (-4.1, -2.9)* | 30.0 (29.2, 30.8) |  | 0.9 (-0.3, 2.2) |
| 2018 | 27.0 (26.2, 27.8) |  | -3.5 (-4.1, -2.9)* | 30.7 (29.9, 31.5) |  | 0.9 (-0.3, 2.2) |
| 2019 | 25.2 (24.5, 26.0) |  | -3.5 (-4.1, -2.9)* | 30.9 (30.0, 31.7) |  | 0.9 (-0.3, 2.2) |

***Indicates that the Annual Percent Change is significantly different from zero at the alpha = 0.05 level.**

##### Table S4: Trends in coronary heart disease mortality rates (2008-2018) among the New Zealand population aged 35-84, estimated by Joinpoint regression model

|  | **Official national mortality data** | | | **GBD estimates** | | |
| --- | --- | --- | --- | --- | --- | --- |
| **Year** | **Age-standardised mortality rates** | **Joinpoint location** | **Annual Percent Change** | **Age-standardised mortality rates** | **Joinpoint location** | **Annual Percent Change** |
| 2008 | 128.2 (123.7, 132.6) |  | -4.6 (-5.0, -4.2)* | 140.7 (135.9, 145.4) |  | -4.4 (-5.2, -3.7)* |
| 2009 | 123.5 (119.2, 127.8) |  | -4.6 (-5.0, -4.2)* | 132.9 (128.4, 137.4) |  | -4.4 (-5.2, -3.7)* |
| 2010 | 118.1 (113.9, 122.3) |  | -4.6 (-5.0, -4.2)* | 126.0121.6, 130.4) |  | -4.4 (-5.2, -3.7)* |
| 2011 | 112.6 (108.6, 116.7) |  | -4.6 (-5.0, -4.2)* | 121.4 (117.2, 125.7) |  | -4.4 (-5.2, -3.7)* |
| 2012 | 108.2 (104.3, 112.1) |  | -4.6 (-5.0, -4.2)* | 116.6 (112.5, 120.7) |  | -4.4 (-5.2, -3.7)* |
| 2013 | 100.1 (96.4, 103.8) |  | -4.6 (-5.0, -4.2)* | 111.4 (107.5, 115.4) | Joinpoint 1 |  |
| 2014 | 97.4 (93.7, 101.0) |  | -4.6 (-5.0, -4.2)* | 109.2 (105.3, 113.1) |  | -2.2 (-5.6, 1.3) |
| 2015 | 94.1 (90.6, 97.7) |  | -4.6 (-5.0, -4.2)* | 106.1 (102.3, 109.9) |  | -2.2 (-5.6, 1.3) |
| 2016 | 86.4 (83.1, 89.8) | Joinpoint 1 |  | 104.9 (101.1, 108.6) | Joinpoint 2 |  |
| 2017 | 89.7 (86.3, 93.0) |  | -1.1 (-4.8, 2.8) | 103.7 (100.0, 107.3) |  | 1.4 (-2.1, 5.1) |
| 2018 | 85.5 (82.2, 88.7) |  | -1.1 (-4.8, 2.8) | 107.9 (104.2, 111.5) |  | 1.4 (-2.1, 5.1) |

***Indicates that the Annual Percent Change is significantly different from zero at the alpha = 0.05 level.**

##### Table S5: Trends in stroke mortality rates (2008-2018) among the New Zealand population aged 35-84, estimated by Joinpoint regression model

|  | **Official national mortality data** | | | **GBD estimates** | | |
| --- | --- | --- | --- | --- | --- | --- |
| **Year** | **Age-standardised mortality rates** | **Joinpoint location** | **Annual Percent Change** | **Age-standardised mortality rates** | **Joinpoint location** | **Annual Percent Change** |
| 2008 | 50.9 (48.1, 53.6) |  | -3.7 (-4.5, -2.9)* | 50.5 (47.7, 53.2) |  | -3.5 (-4.7, -2.3)* |
| 2009 | 49.8 (47.1, 52.5) |  | -3.7 (-4.5, -2.9)* | 48.1 (45.4, 50.7) |  | -3.5 (-4.7, -2.3)* |
| 2010 | 47.0 (44.4, 49.6) |  | -3.7 (-4.5, -2.9)* | 46.0 (43.4, 48.6) |  | -3.5 (-4.7, -2.3)* |
| 2011 | 47.2 (44.6, 49.8) |  | -3.7 (-4.5, -2.9)* | 45.0 (42.4, 47.5) |  | -3.5 (-4.7, -2.3)* |
| 2012 | 43.2 (40.7, 45.6) |  | -3.7 (-4.5, -2.9)* | 43.5 (41.1, 46.0) |  | -3.5 (-4.7, -2.3)* |
| 2013 | 39.8 (37.5, 42.2) |  | -3.7 (-4.5, -2.9)* | 41.8 (39.4, 44.3) | Joinpoint 1 |  |
| 2014 | 43.4 (41.0, 45.9) |  | -3.7 (-4.5, -2.9)* | 41.9 (39.5, 44.3) |  | -0.9 (-2.1, 0.3) |
| 2015 | 40.6 (38.3, 42.9) |  | -3.7 (-4.5, -2.9)* | 41.0 (38.7, 43.4) |  | -0.9 (-2.1, 0.3) |
| 2016 | 35.7 (33.5, 37.8) |  | -3.7 (-4.5, -2.9)* | 40.1 (37.8, 42.4) |  | -0.9 (-2.1, 0.3) |
| 2017 | 36.8 (34.7, 39.0) |  | -3.7 (-4.5, -2.9)* | 39.5 (37.3, 41.7) |  | -0.9 (-2.1, 0.3) |
| 2018 | 35.3 (33.3, 37.4) |  | -3.7 (-4.5, -2.9)* | 40.9 (38.7, 43.1) |  | -0.9 (-2.1, 0.3) |

***Indicates that the Annual Percent Change is significantly different from zero at the alpha = 0.05 level**
